# Multiple imputation to quantify misclassification in observational studies of the cognitively impaired: an application for pain assessment in nursing home residents

**DOI:** 10.1186/s12874-021-01327-5

**Published:** 2021-06-26

**Authors:** Anthony P. Nunes, Danni Zhao, William M. Jesdale, Kate L. Lapane

**Affiliations:** Division of Epidemiology, Department of Population and Quantitative Health Sciences, University of Massachusetts Medical School, 368 Plantation Street, Worcester, MA 01605 USA

**Keywords:** Misclassification, Multiple imputation, Pain, Nursing homes

## Abstract

**Background:**

Despite experimental evidence suggesting that pain sensitivity is not impaired by cognitive impairment, observational studies in nursing home residents have observed an inverse association between cognitive impairment and resident-reported or staff-assessed pain. Under the hypothesis that the inverse association may be partially attributable to differential misclassification due to recall and communication limitations, this study implemented a missing data approach to quantify the absolute magnitude of misclassification of pain, pain frequency, and pain intensity by level of cognitive impairment.

**Methods:**

Using the 2016 Minimum Data Set 3.0, we conducted a cross-sectional study among newly admitted US nursing home residents. Pain presence, severity, and frequency is assessed via resident-reported measures. For residents unable to communicate their pain, nursing home staff document pain based on direct resident observation and record review. We estimate a counterfactual expected level of pain in the absence of cognitive impairment by multiply imputing modified pain indicators for which the values were retained for residents with no/mild cognitive impairment and set to missing for residents with moderate/severe cognitive impairment. Absolute differences (∆) in the presence and magnitude of pain were calculated as the difference between documented pain and the expected level of pain.

**Results:**

The difference between observed and expected resident reported pain was greater in residents with severe cognitive impairment (∆ = -10.2%, 95% Confidence Interval (CI): -10.9% to -9.4%) than those with moderate cognitive impairment (∆ = -4.5%, 95% CI: -5.4% to -3.6%). For staff-assessed pain, the magnitude of apparent underreporting was similar between residents with moderate impairment (∆ = -7.2%, 95% CI: -8.3% to -6.0%) and residents with severe impairment (∆ = -7.2%, 95% CI: -8.0% to -6.3%). Pain characterized as “mild” had the highest magnitude of apparent underreporting.

**Conclusions:**

In residents with moderate to severe cognitive impairment, documentation of any pain was lower than expected in the absence of cognitive impairment. This finding supports the hypothesis that an inverse association between pain and cognitive impairment may be explained by differential misclassification. This study highlights the need to develop analytic and/or procedural solutions to correct for recall/reporter bias resulting from cognitive impairment.

**Supplementary Information:**

The online version contains supplementary material available at 10.1186/s12874-021-01327-5.

## Background

Pain, the “fifth vital sign”, is underassessed and undertreated across the spectrum of care [1]. Pain assessment is inherently dependent on communication, either explicitly through verbal communication or via implicit signs and behaviors consistent with pain (e.g., moaning, flinching). For this reason, misclassification of pain may result from misreporting, or by unobserved or unrecognized signs of pain.

The importance of accurate assessment of pain in the nursing home setting is amplified due to the high prevalence of chronically present painful comorbidities. Pain is commonly experienced by nursing home residents and is a critical metric of quality of care [2]. Older adults in nursing homes are more likely to experience prolonged pain from multiple sources, resulting from diseases like arthritis, wound or injury healing, and disability [3, 4]. In nursing home residents, pain affects measures of well-being [5] including depression, anxiety, sleep disturbance, social isolation, and immobility [6, 7].

Despite its prevalence and impact on the lives of residents, pain is often under-recognized in nursing homes [8, 9] in part because residents often have communication challenges [10] and/or cognitive impairment [11]. Eight percent of all residents are unable to self-report pain [12]; two thirds of residents have cognitive impairment or dementia [13]. Residents with cognitive impairment are less likely to have pain documented by self-report [14], and for those with staff-assessed pain, are less likely to have typical external manifestations of pain documented [14].

Previously, we have shown that residents with cognitive impairment are less likely to have pain documented and are less likely to have pain management strategies used. This is in contrast to literature concluding that pain sensitivity is unaltered or possibly modestly elevated in those with cognitive impairments [15]. While misclassification may be a contributing factor to the observed lower estimates of pain prevalence in residents with moderate and severe cognitive impairment, prior studies have not attempted to isolate the impact of misclassification from true differences in the pain experience of nursing home residents of differing cognitive functioning levels. This study addresses this research gap by asking the question: “What levels of pain would nursing home residents with moderate-to-severe cognitive impairment have documented in the absence of their cognitive impairment?” This question implicitly recognizes a counterfactual realty in which residents with cognitive impairment could be observed in the absence of their cognitive impairment. Because this cannot be observed in the real world, the counterfactual observation is always missing. Thus, missing data methods conceptually align with the goals of addressing this question. Using an extension of multiple imputation methods, we sought to provide estimates of the absolute magnitude of misclassification of pain, pain frequency, and pain intensity by level of cognitive impairment. Further, we estimate the extent of the difference in pain misclassification by key sociodemographic variables. We hypothesized that pain would be under-reported in those with cognitive impairment, and with greater under-reporting in those with severe cognitive impairment.

## Methods

### Data source and population

We conducted a cross-sectional study among nursing home residents who were newly admitted to US nursing homes in 2016, as identified in the Minimum Data Set 3.0 (MDS 3.0) [16]. The MDS assessment is a federally required screening and assessment tool used by all nursing home facilities certified to participate in Medicare and/or Medicaid (> 96% of all US nursing homes). The MDS comprehensive admission assessment includes resident demographic and clinical characteristics, cognitive and physical functioning levels, indices of pain, mood disorders, and other co-morbidities. The assessments are conducted by registered nurses at each nursing home facility and includes information gained by interviewing residents and their caregivers.

We included residents aged ≥ 50 years. If a resident had multiple admissions in 2016, we selected their first admission for inclusion in this study. We excluded residents who: 1) were in comatose status; 2) did not have valid cognitive function information; or 3) lacked a valid response to at least one of the pain items. Residents with at least one valid response to pain items were included in the overall study, but excluded from item-specific analyses if they lacked a valid response to the item of interest.

### Assessment of cognitive impairment

Resident cognitive function was measured with the Brief Interview for Mental Status (BIMS) or the Cognitive Performance Scale (CPS). The BIMS is a seven-item scale that assesses resident cognitive function in three domains: repetition of three words, temporal orientation, and recall, yielding a total score between 0–15, with higher scores indicating less cognitive impairment. If residents could not answer the BIMS, the CPS was completed. The CPS includes five MDS items examining resident comatose status, short-term memory, ability to be understood by others, daily decision-making capacity, and independence with eating. The CPS ranges from 0–6, with higher score suggesting more cognitive impairment. Resident cognitive function was categorized into three levels based on their BIMS or CPS score, according to the Nursing Home Compendium definition [17]: no/ mild impairment (BIMS = 13–15; CPS = 0–2), moderate impairment (BIMS = 8–12; CPS = 3–4) and severe impairment (BIMS = 0–7; CPS = 5–6).

### Assessment of pain

For pain items, residents are first asked to self-report their symptoms. If they cannot answer, staff-assessed pain is conducted [18]. Specifically, residents were asked to describe the pain they experienced in the past 5 days in the following domains: pain presence, pain frequency, pain effect on function, and pain intensity. For pain intensity, residents were advised to either rate their pain on a 0–10 scale, with 0 being no pain and 10 being the most horrible pain they could imagine or to verbally describe the intensity of the pain as mild, moderate, severe, or very severe/horrible. Some residents completed both (*n* = 20,183, 1.05%) and for the purposes of this study, all information was used. If nursing home staff determined that residents could not complete the self-reported assessments of pain, staff-assessed measures are used. For pain, nurses complete the MDS with information gleaned from an examination of the resident medical records, interviews of staff in direct contact with the resident, and direct observations of the resident. Based on information from these multiple sources, the nurse infers whether the resident experienced pain in the past 5 days. Nurses document indicators for possible pain, including non-verbal sounds, vocal complaints of pain, facial expressions, or protective body movements. If any of these pain behaviors were documented, they proceed to rate their frequency (1–2 days, 3–4 days or daily in the past 5 days).

### Covariates

Based on prior literature, we identified a list of risk factors for pain including demographic factors (e.g., sex, age, race/ethnicity), types of admission (e.g., post-acute care), physical functioning, and comorbidities (e.g., cancer, heart failure, cirrhosis, pneumonia, diabetes mellitus, arthritis, fractures, Alzheimer’s disease or dementia, anxiety disorder, and pulmonary diseases). Physical functioning was measured using the Activity of Daily Living (ADL) Hierarchy Scale [19]. The ADL score uses four MDS items assessing resident performance on self-hygiene, toilet use, locomotion on unit, and eating, yielding a total score of 0–6. Based on the ADL Hierarchy score, residents were further categorized into no/ minimal physical impairment (0–2), moderate impairment (3–4), and severe impairment (5–6). To ascertain the presence of a comorbidity, nurses would first refer to a resident’s medical records to assess whether a physician-documented diagnosis of the condition existed. If a diagnosis was documented, nurses would further assess whether the condition affected the resident over the past 7 days. The MDS 3.0 includes assessments of individual components of the Patient Health Questionnaire-9 (PHQ-9 or PHQ10/OV) over the past 2 weeks and their frequency of occurrence (never or 1 day; 2–6 days; 7–11 days or 11–14 days) [20].

### Analysis

Our analysis was conceptually designed around the counterfactual question, “What levels of pain would nursing home residents with moderate-to-severe cognitive impairment have documented in the absence of cognitive impairment?” In addition to quantifying the magnitude of misclassification, we aimed to generate individual-level corrected indicators of pain.

**Fig. 1:**
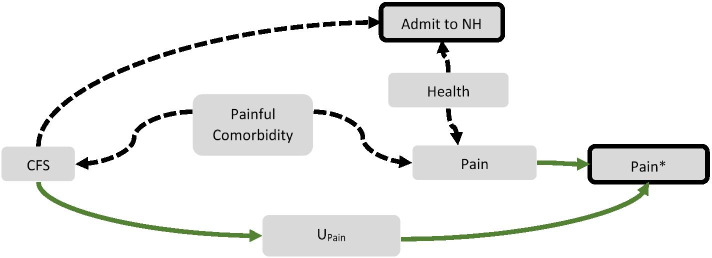
Hypothesized causal paths between cognitive functioning status (CFS) and documented pain (Pain*). CFS may be associated with pain via a common cause or nursing home selection forces, represented by the dashed lines. In this study, we are interested in the potential bias through differential misclassification (U_Pain_) as represented by the solid green arrows

We constructed a directed acyclic graph [21, 22] shown in Fig. 1 to assess potential paths between cognitive functioning and documented pain, including via misclassification. Under this hypothesized model, the association between cognitive impairment and pain may be through confounding paths, selection forces, and differential pain sensitivity. Though pain sensitivity is a hypothetical causal node on the path between cognitive impairment and pain, the existing literature suggests that there are generally no alterations in pain sensitivity associated with cognitive impairment [15]. In other words, in the absence of confounding or bias, holding all else constant, individuals with cognitive impairment should have similar pain experiences as compared to those without cognitive impairment. Recall/observation bias is introduced through our dependence on pain assessments rather than a hypothetical objective detection of pain. The focus of this analysis was to assess the extent to which misclassification results in an under ascertainment of pain. Our imputation approach explicitly treats the counterfactual as a missing data problem in which the cognitively intact estimate of pain is missing in those with moderate to severe cognitive impairment.

By comparing the estimated counterfactual level of pain to the level of pain documented on the MDS 3.0 in residents with cognitive impairment, we estimate the magnitude of potential information bias. To operationalize this approach, we rely on a multiple imputation method in which we impute counterfactual values of pain in residents with moderate-to-severe cognitive impairment. Details are provided in the Methods Supplement. Briefly, this process included two steps: (1) creation of imputable variables representative of the counterfactual constructs (i.e., pain reported in the absence of cognitive impairment), and (2) multiple imputation via fully conditional specification of the counterfactual constructs of pain among those with moderate-to-severe cognitive impairment.

We first created imputable variables corresponding to each individual item in resident-assessed pain measures, based on the resident’s level of cognitive impairment. For residents with no or mild cognitive impairment, the values of the imputable variables retained that of the corresponding items in resident-reported pain measures. For residents with moderate to severe cognitive impairment, the imputable variables were all set to missing. Next, we created two combined imputable variables for the presence of pain and pain frequency accounting for both resident-reported and staff-assessed measures. For residents with no or mild cognitive impairment, the values of the combined imputable variables were set to the values of the resident-reported pain measures. When resident-reported pain measures were not available, the values of the combined imputable variables were set to the corresponding staff-assessed variable. Similarly, we set the values of the combined imputable variables to missing for residents with moderately or severely impaired cognitive functioning. For example, if a resident was unable to complete the self-reported pain questions, but the nurse observed non-verbal sounds indicating the presence of possible pain, the imputable variable for the presence of pain would be equal to 1 if the resident was cognitive intact or mild impaired and missing if their cognitive impairment was moderate or severe.

Imputations were conducted with SAS software (Version 9.4, SAS Institute Inc., Cary, NC, USA). Because the variables included binary, multinomial, and continuous response options, we implemented a fully conditional specification imputation [23], using the discriminant function to impute categorical variables. To select covariates to be included in the multiple imputation, we relied on substantive knowledge (Directed Acyclic Graphs and a-priori covariates: Activities of Daily Living Score, potentially painful medical conditions) and preliminary analyses (covariate distributions, and exploratory stepwise selection models). Exploratory stepwise selection models were conducted with pain indicators as the dependent variable to evaluate candidate variables to include in the MI models from the broader set of MDS variables relying on (1) AIC minimization and (2) p-value selection with an inclusion criterion set to 0.15. The full list of covariates included in the MI model is provided in Methods Supplement. To improve run-time efficiency, imputations were conducted in parallel for each state. We generated 50 imputations to enable assessment of between-imputation variance.

Distributions of resident characteristics were calculated by levels of cognitive impairment. We calculated frequencies and proportions for categorical variables, and median, 25^th^ and 75^th^ percentiles for continuous variables. In addition, distributions of MDS 3.0 recorded pain indicators were assessed by level of cognitive impairment.

We calculated the difference between MDS 3.0 documented pain and multiply imputed pain. Under the assumption that imputed pain represents the counterfactual assessment of pain in the absence of cognitive impairment, we interpret the calculated difference as an estimate of potential information bias. The calculated delta for a resident represents the difference between their documented pain and an estimate of pain corresponding to a cognitively intact but otherwise identical resident. By accounting for demographic and clinical characteristics when assessing the difference between observed and expected pain metrics, our estimated differences quantify the impact of cognitive impairment on documentation of pain among newly admitted nursing home residents. We report the mean absolute difference between observed and expected values (∆) and 95% confidence intervals (CI), accounting for within- and between-imputation variance through Rubin’s Rules for a pooled MI variance, as estimated in SAS MI Analyze. Estimates are provided separately for residents with moderate and severe cognitive impairment, and by resident gender, pain management (scheduled + *pro re nata* (PRN), scheduled only, PRN only, no pharmacological pain management), fracture, and arthritis. In a large population such as this, statistical inference from p-values has limited utility. We considered absolute differences of 5% or greater to be notable.

## Results

### Study population

**Fig. 2:**
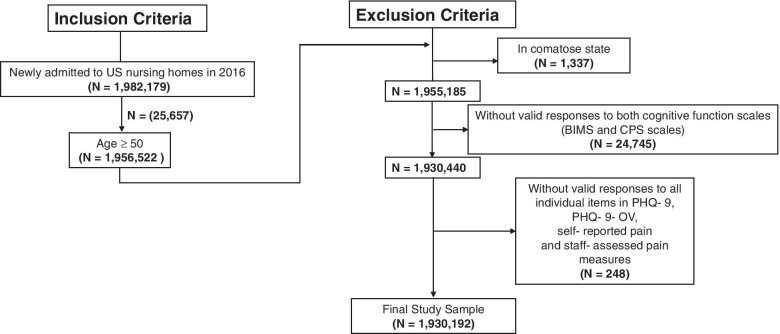
Flowchart of Study Sample Selection

Figure 2 shows the flowchart for the study population. Our final study sample consisted of 1,930,192 residents.

### Distribution of resident characteristics

Relative to residents with no/mild cognitive impairment, those with moderate or severe cognitive impairment were older, with the proportion aged ≥ 85 years increasing from a quarter in those with no/mild impairments to half among those with severe cognitive impairment (Table 1). With the exception of neurologic diseases, the prevalence of chronic diseases (including cancer, heart, circulatory, gastrointestinal, metabolic, and pulmonary diagnoses) tended to decline with increasing cognitive impairment. In contrast, the prevalence of dementias increased from 7.7% among residents with no/mild cognitive impairment to 62.0% among those with severe cognitive impairment.

**Table 1:** Characteristics of newly admitted nursing home residents by level of cognitive impairment (*n* = 1,930,192)

Characteristic	Level of Cognitive Impairment		
	No/Mild, % (*n* = 1,160,053)	Moderate, % (*n* = 430,442)	Severe, % (*n* = 339,697)
Age, years			
50–64	10.9	5.7	4.2
65–74	29.0	17.9	13.5
75–84	33.3	33.3	32.2
85 +	26.7	43.0	50.1
Women	62.7	58.0	61.4
Race/ethnicity: Non-Hispanic, White	81.1	78.2	75.3
Non-Hispanic, Black	9.6	10.8	12.2
Hispanic or Latino of any race(s)	3.7	4.8	5.8
Non-Hispanic American Indian, Alaskan Native, Asian, Native Hawaiian, Pacific Islander, multiracial	1.9	2.4	3.0
Marital Status:			
Never married	12.1	10.4	9.8
Married	33.4	32.4	32.8
Widowed	36.1	42.3	44.7
Separated	1.1	0.9	0.8
Divorced	13.3	10.2	8.1
Activities of daily living			
Moderate limitations	68.1	68.4	63.5
Dependent	12.9	19.0	28.2
Diagnoses			
Cancer	10.7	10.1	8.3
Heart/Circulatory			
Heart Failure	22.2	22.5	17.6
Coronary Artery Disease	24.5	25.0	21.7
Venous Thromboembolism	3.9	3.4	3.0
Peripheral Vascular/Arterial Disease	7.8	7.2	5.6
Gastrointestinal			
Cirrhosis	1.3	1.1	0.7
GERD or Ulcer	35.5	33.0	29.6
Inflammatory Bowel Disease/ Ulcerative Colitis	1.4	1.1	0.9
Infections			
Urinary Tract Infection (last 30 days)	10.0	13.5	14.9
Pneumonia	7.9	9.0	8.5
Metabolic			
Diabetes	35.6	32.4	27.9
Thyroid Disorder	22.2	21.8	21.7
Musculoskeletal			
Arthritis	28.6	23.4	20.8
Osteoporosis	8.9	9.0	9.5
Fracture (hip and other)	19.1	17.7	16.4
Neurological/Psych			
Dementia/Alzheimer’s	7.7	32.3	62.0
Anxiety disorder	19.4	19.2	20.5
Depression	29.5	31.8	32.0
Pulmonary			
Asthma/COPD	26.7	22.7	16.7
Respiratory Failure	5.2	4.6	3.9

### Distribution of documented pain indicators

The percent with any documented pain among those with severe cognitive impairments (staff-assessed: 36.2%; resident-reported: 34.0%) was approximately half of the documented pain in those with no/mild cognitive impairment (staff-assessed: 50.8%; resident-reported: 66.0%). Similar differences were seen in the resident-reported metrics of pain severity and frequency (Table 2). The presence of pain management also declined with increasing cognitive impairment. Overall, residents with cognitive impairment were less likely to receive pain management (53.7%) as compared to those with no/mild cognitive impairment (76.4%). Receipt of scheduled and PRN pain management was observed in 13.6% of residents with severe cognitive impairment and 28.0% of residents with no/mild cognitive impairment.

**Table 2:** Distribution of pain indicators among newly admitted nursing home residents, by level of cognitive impairment (*n* = 1,930,192)^a^

Pain Indicators	Level of Cognitive Impairment					
	No/Mild, % (*n* = 1,160,053)		Moderate, % (*n* = 430,442)		Severe, % (*n* = 339,697)	
	n	%	n	%	n	%
Staff-Assessed Pain	38,018		33,967		722,310	
Yes		50.8		42.0		36.2
No		49.2		58.0		63.8
Resident-Reported Pain	1,117,134		397,072		271,192	
Yes		66.0		49.4		34.0
No		34.0		50.6		66.0
Resident-reported pain details^b^						
Pain Frequency	1,115,210		394,128		265,555	
No pain		34.1		51.0		67.4
Rarely		4.9		5.0		4.1
Occasionally		34.2		27.0		19.3
Frequently		21.0		13.3		7.4
Almost Constantly		5.8		3.7		1.8
Pain Numeric Rating	942,528		338,813		232,527	
Median (25^TH^,75^TH^ %)		4 (0–6)		0 (0–5)		0 (0–0)
Pain Verbal Descriptor Scale	564,584		258,391		212,453	
No pain		67.3		77.7		84.2
Mild		11.9		9.1		7.1
Moderate		17.4		11.0		7.3
Severe		3.1		2.0		1.3
Very Severe		0.3		0.2		0.2
Pharmacologic Pain Management	1,159,468		430,198		339,472	
Scheduled + PRN		28.0		19.3		13.6
Scheduled Only		8.0		10.0		11.7
PRN Only		40.5		34.5		28.5
None		23.6		36.3		46.3

^a^Residents with at least one valid response to pain items were included in the overall study, but excluded from item-specific analyses if they lacked a valid response to the item of interest. As such, we present the sample size specific to each estimate

^b^For resident-reported details, values were set to “no pain” if explicitly documented as “no pain” or if missing and staff-assessed or resident-reported pain was “no pain”

### Pain differences

Comparing documented pain to the expected level of pain in the absence of cognitive impairment, we observed consistent underreporting (Table 3). The magnitude of the difference between observed minus expected for staff-reported any pain was similar between those with moderate impairment (∆ = -7.2%, 95% CI: -8.3% to -6.0%) and those with severe impairment (∆ = -7.2%, 95% CI: -8.0% to -6.3%). When relying on resident reported pain, our estimate of pain underreporting was greater in residents with severe cognitive impairment (∆ = -10.2%, 95% CI: -10.9% to -9.4%) than residents with moderate cognitive impairment (∆ = -4.5%, 95% CI: -5.4% to -3.6%). In addition to estimated underreporting of pain, metrics of pain frequency and severity appeared to be underreported. Though pain appeared to be underreported across all levels of severity, pain characterized as “mild” had the highest estimated underreporting.

**Table 3:** Differences between observed pain responses and imputed pain responses among nursing home residents with moderate or severe cognitive impairment (*n* = 770,139)

Pain	Moderate Cognitive Impairment		Severe Cognitive Impairment	
	n	∆ (95% Confidence Interval (CI))	N	∆ (95% CI)
Any Pain				
Staff-Assessed	33,967	-7.2 (-8.3 to -6.0)	72,231	-7.2 (-8.0 to -6.3)
Resident-reported	397,072	-4.5 (-5.4 to -3.6)	271,192	-10.2 (-10.9 to -9.4)
Resident-reported pain details^a^				
Pain Frequency	394,128		265,555	
No pain		4.8 (3.9 to 5.6)		11.0 (10.2 to 11.7)
Rarely		-2.0 (-2.8 to -1.2)		-2.0 (-2.8 to -1.2)
Occasionally		3.8 (2.4 to 5.2)		-0.0 (-1.2 to 1.2)
Frequently		-2.1 (-3.5 to -0.7)		-4.4 (-5.6 to -3.3)
Almost Constantly		-4.4 (-6.0 to -2.9)		-4.5 (-5.8 to -3.2)
Pain Numeric Rating Scale	338,813	-0.8 (-0.9 to -0.7)	232,527	-1.2 (-1.3 to -1.1)
Pain Verbal Descriptor Scale	258,391		212,453	
No pain		17.0 (16.3 to 17.7)		18.9 (18.3 to 19.6)
Mild		-6.0 (-7.2 to -4.9)		-6.6 (-7.7 to -5.5)
Moderate		-1.8 (-2.8 to -0.7)		-3.9 (-4.9 to -2.9)
Severe		-4.3 (-5.1 to -3.5)		-4.1 (-5.9 to -3.4)
Very Severe		-4.9 (-5.9 to -3.8)		-4.3 (-5.2 to -3.3)

∆: Absolute difference, calculated as the mean value of observed – expected. For categorical variables, converted to percentile by multiplying difference by 100. For the pain numeric rating scale calculated as observed rating-expected rating

^a^For resident-reported details, values were set to “no pain” if explicitly documented as “no pain” or if missing and staff-assessed or resident reported any-pain was “no pain”

### Pain differences by resident characteristics

Table 4 shows the estimated association between cognitive impairment and underreporting of pain by strata of resident gender, arthritis, fracture, and pharmacologic pain management. Though similar in magnitude, the association was slightly larger in women than men. When stratified by pharmacologic pain management, estimated underreporting of pain was notably higher among those without scheduled pain medications.

**Table 4:** Differences between expected and observed any pain responses among nursing home residents with moderate or severe cognitive impairment, stratified by demographic variables (*n* = 770,139)

Strata	Moderate Cognitive Impairment		Severe Cognitive Impairment	
	n	∆ (95% Confidence Interval (CI))	n	∆ (95% CI)
Gender				
Women	248,483	-5.3 (-6.2 to -4.4)	207,577	-10.8 (-11.6 to -10.0)
Men	179,669	-4.3 (-5.2 to -3.5)	130,294	-9.0 (-9.7 to -8.2)
Pain Rx				
Scheduled + PRN	82,455	-10.2 (-11.5 to -8.8)	45,800	-20.3 (-21.5 to -19.0)
Scheduled Only	42,559	13.7 (12.5 to 15.0)	39,434	3.6 (2.3 to 4.8)
PRN Only	147,510	-15.0 (-16.3 to -13.8)	96,105	-25.7 (-26.9 to -24.4)
None	155,432	2.4 (2.0 to 2.9)	156,357	-1.0 (-1.5 to -0.5)
Fracture				
Yes	75,901	-4.4 (-5.5 to -3.3)	55,280	-11.6 (-12.7 to -10.6)
No	352,197	-5.0 (-5.8 to -4.1)	282,560	-9.8 (-10.5 to -9.1)
Arthritis				
Yes	99,964	-4.7 (-5.5 to -3.7)	70,274	-11.2 (-12.1 to -10.4)
No	328,132	-4.9 (-5.8 to -4.1)	267,560	-9.8 (-10.6 to -9.1)

∆: Absolute difference in percentage between calculated as percentage observed – percentage expected

## Discussion

Concerns about differential misclassification of pain arise from prior evidence of less frequently documented pain in residents with cognitive impairment, and the plausibility of underreporting due to communication or recall limitations associated with cognitive impairment. Epidemiologists are well trained to devise and apply measurement error mitigation methods or sensitivity analyses [24, 25]. To further understand the potential magnitude of pain misclassification, this study design and methods were motivated by our counterfactual causal question [26]: “Among residents with cognitive impairment, what is the difference between what was actually reported and what residents would have reported if they were cognitively intact?” Long-established multiple imputation techniques estimate a set of proxies for an unmeasured gold standard, generally reducing bias and loss of precision relative to model-based correction methods [27, 28]. The multiple imputation approach enabled us to include a large number of demographic and clinical correlates of pain. We observed that residents with moderate or severe cognitive impairments were less likely to have documentation of any pain present as compared to their expected level of pain in the absence of cognitive impairment. Likewise, the magnitude of observed pain severity and frequency among residents with cognitive impairment was lower than expected. Though underreporting or under recognition of pain was most notable in those with severe cognitive impairment and when resident-reported, there was substantial estimated underreporting in staff-assessed pain, and in those with moderate cognitive impairment. Estimated underreporting of pain in both staff-assessed and resident-reported pain metrics suggests that cognitive impairment may impact recall of recent pain, the ability to communicate pain symptoms, or the manifestation of typical pain behaviors.

This study supports the hypothesis that cognitive impairment is associated with measurement error of resident-reported and staff-assessed metrics of pain. Given the prevalence of cognitive impairment in the nursing home population, and the magnitude of the underestimation of pain observed in this study, the impact of cognitive impairment on pain measurement has clinical implications. To be adequately treated, pain must be recognized. In older adults, inadequately treated pain has been linked to low quality of life [29, 30] and disruptive behaviors [31]. Techniques to improve pain assessment in those with severe cognitive impairment have included combining observations with resident-reported information [32–34]. For such approaches to work in nursing homes, barriers to pain assessment including time constraints and training must be addressed [35, 36].

The implications of our study findings on research must also be considered. Studies of effectiveness, tolerability, and quality of pain management strategies in nursing home residents may also be susceptible to bias caused by errors in pain measurement. We previously reported that residents with severe cognitive impairment were more likely to have untreated or undertreated pain [37, 38]. Accounting for the underestimate of pain we observe in this study, we anticipate that correcting for pain measurement error due to cognitive impairment would result in a notably larger association with untreated and undertreated pain. To address measurement concerns, research on the long-term effects of analgesics on health outcomes has excluded cognitively impaired residents [39]. Potential solutions to this measurement issue are needed to generate evidence in a population often neglected by research. Our approach acknowledges that documented pain is a factor in resident pain experience, their ability to communicate their pain, and the ability of nursing home staff to assess and record the signs and symptoms of pain. For research (and clinical) purposes, we are principally interested in the component of documented pain due to the actual pain experience, removing the effect due to resident ability to communicate and staff ability to assess and record their pain. Assuming the potential for reporting and documentation bias is reduced in cognitively intact residents, then approaches including multiple imputation, prediction methods, and simple correction factors may be useful. In future studies, we will explore and compare correction strategies ranging from machine learning prediction to stratum specific correction factors.

Counterfactual prediction is a common approach implemented in epidemiology, most often for the purpose of causal inference [40]. The weighting and matching methods employed in marginal structural models, inverse probability of treatment weighting, and propensity score matching are each methods that employ counterfactual prediction. These methods excel in quantifying differences between a factual and counterfactual group to quantify an estimate of a causal association. Thus, these methods would be suitable approaches to enable an estimation of the effect of cognitive impairment on documented pain. Multiple imputation and g-formula estimation have also been proposed as alternative solutions for counterfactual prediction [41]. We employed a multiple imputation model because of our framing of the question as a counterfactual missing data problem, our desire to select a method that was intuitive and readily available, and our interest in generating an individual-level corrected value that may be utilized in subsequent analyses. Multiple imputation is an established method fulfilling each of our driving motivations. In particular, the direct generation of corrected measures of pain available at the individual resident level afforded multiple imputation an advantage over weighting and matching based approaches.

While we have attempted to reduce the potential for other sources of bias when quantifying the magnitude of pain measurement error, there are inherent limitations to the data that limit our ability to draw a definitive conclusion. With respect to our imputations, the primary concerns are (1) unmeasured confounders, (2) differential misclassification of observed determinants of pain, and (3) true differences in pain sensitivity by cognitive functioning status. Though the MDS assessments include detailed measures of functional status and active medical conditions, pain has a broad spectrum of determinants that may be difficult to ascertain. We assessed the potential impact of unmeasured confounders through e-value estimation, where the e-value is the necessary magnitude of association (relative risk conditioned on observed covariates) between an unmeasured confounder and pain and cognitive impairment, that could fully explain the observed differences between documented pain and imputed counterfactual pain [42]. The e-value for our self-reported pain indicator was 1.92 for severe cognitive impairment and 1.09 for moderate cognitive impairment. For staff assessed pain, the e-value was 1.62 for severe and mild cognitive impairment. Just as cognitive impairment impacts assessments of pain, it may also influence the accuracy of the clinical diagnoses and measures that were included in the imputation model. This may be more problematic for subjective assessments. It is plausible that cognitive impairment results in measurement error of active diagnoses, either through differential screening and documentation or through consideration of what is interpreted as an “active” diagnosis. The multiple imputation method does not account for differential misclassification of these diagnoses, to the extent such misclassification is not correlated with other included imputation variables. If the covariates included in the imputation model are differentially misclassified with respect to cognitive impairment, then the estimated pain in the absence of cognitive impairment may also be biased. In addition to the measurement limitations, we note that the differences in the counterfactual pain and documented pain may reflect a combination of information bias and residual differences in experience even after accounting for demographic and clinical factors. That is, the mechanism leading to cognitive impairment may also impact pain sensation and reaction. Though existing evidence suggests that this mechanism would have a minimal impact, there is some evidence that pain sensation modestly increases in some etiologies of cognitive impairment, potentially resulting in an imputed counterfactual that underestimates true pain. We conducted a quantitative bias analysis under the assumption that our analysis resulted in a lower sensitivity prediction of pain among residents with cognitive impairment [43]. In this scenario, the quantitative bias analysis suggests that our conclusions would be robust to the resulting bias, with a possibility of underestimating the true difference. We also explored the potential for bias under the unexpected scenario that imputed pain is overestimated (e.g., for self-reported pain and severe cognitive impairment, with a sensitivity of 0.9 for imputed pain, the specificity would need to be less than 0.8 to explain our observed differences). These results provide some confidence that our findings are robust to, though not immune from, plausible scenarios of inaccuracies in our counterfactual imputation.

## Conclusion

Accurately characterizing the presence and severity of pain in nursing home residents is critical for the evaluation of resident outcomes and quality of care. In residents with moderate to severe cognitive impairment, we observed that the documentation of any pain was significantly (statistically and clinically) lower than expected in the absence of cognitive impairment. Likewise, pain severity and frequency were underestimated in residents with moderate to severe cognitive impairment. This has notable implications on pain management and research in nursing home populations. By documenting the direction and magnitude of pain misclassification, this study will inform the conduct and interpretation of pain-based research in nursing home residents. Furthermore, it highlights the need to develop analytic and/or procedural solutions to correct for pain misclassification due to cognitive impairment.

## Supplementary Information

**Additional file 1: **Methods overview. **Supplemental Table 1.** Listing of covariates included in the multiple imputation of pain metrics. SAS code for multiple imputation.

## Data Availability

We gained access to the data via a Data Use Agreement with the Centers for Medicaid and Medicare Services. In the Methods Appendix we provide the code used to generate analyses.

## Abbreviations

ADL: Activity of Daily Living
BIMS: Brief Interview for Mental Status
CPS: Cognitive Performance Scale
MDS: Minimum Data Set
PHQ-9 or PHQ10/OV: Patient Health Questionnaire-9
PRN: *Pro re nata*
