# Supplementary material for: Multiple imputation to quantify misclassification in observational studies of the cognitively impaired: an application for pain assessment in nursing home residents
Source: BMC Med Res Methodol. 2021 Jun 26;21:132. doi: 10.1186/s12874-021-01327-5 (PMC8235835; doi:10.1186/s12874-021-01327-5)
Supplement: Supplementary file 1 — Additional file 1: Methods overview. Supplemental Table 1. Listing of covariates included in the multiple imputation of pain metrics. SAS code for multiple imputation. [file 12874_2021_1327_MOESM1_ESM.docx]

**Supplemental File**

**METHODS OVERVIEW**

**Analytic Motivation:**

Comparing the pain experience between those with and without cognitive impairment cannot be used as an approximation of under-reporting of pain due to cognitive impairment in nursing home residents. As depicted in Figure 1, cognitive impairment and painful medical events/conditions are independent causes of nursing home admission. By restricting to a nursing home resident population, we have thus conditioned on a collider that will introduce a negative association between cognitive impairment and pain. Other conditions may act as a common cause of cognitive impairment and pain. Thus, we must account for medical conditions that are a common cause between nursing home admissions and pain (on the collider path) and between cognitive impairment and pain (on the confounding path). The large number of potential covariates necessitates an alternative to multivariable adjustment.

**Approach:** When evaluating analytic approaches, we opted to employ a multiple imputation approach because (1) it aligns with our framework that the counterfactual observation of pain in the absence of cognitive impairment is a missing data problem and (2) it is amenable to high dimensionality resulting from the large number of potential covariates. To operationalize this approach, we impute the counterfactual values of pain in residents with moderate-to-severe cognitive impairment through two steps: (1) creation of imputable variables representative of the counterfactual constructs (i.e., pain reported in the absence of cognitive impairment), and (2) multiple imputation via fully conditional specification of the counterfactual constructs of pain among those with moderate-to-severe cognitive impairment.

**Creation of Imputable Variables:** The imputable variables were defined as the observed pain indicators in the absence of cognitive impairment. Thus, these indicators were considered observed among residents with no/mild cognitive impairment and unobserved in residents with moderate and severe cognitive impairment. To create these variables, for each pain indicator we set the value of the new imputable variable to be equal to the documented value pain. If residents were categorized as having moderate or severe cognitive impairment, we then deleted the value of pain in the imputable variable, setting the value as “missing” or “.”. The end result of this process was a dataset containing the unaltered pain indicators and a corresponding set of recoded pain indicators representing pain in the absence of cognitive impairment. Because measures of mood and depression were collected via a similar approach (combination of resident resident-reported and staff assessment) and potentially susceptible to a comparable bias, an analogous approach was implemented for the pain and mood indicators (i.e., the PHQ-9 assessments). When implementing the imputation, the unaltered pain and mood indicators were excluded from the model.

Imputations were conducted relying on the Proc MI procedure available in SAS (Version 9.4, SAS Institute Inc., Cary, NC, USA). Because the variables included binary, multinomial, and continuous response indicators, we implemented a fully conditional specification imputation,[24] using the discriminate function to impute the categorical variables. Continuous and binary covariates and the continuous pain indicator (pain numeric rating scale) were included as continuous variables, thus assuming a multivariate normal distribution of covariates. Categorical pain indicators included resident-reported pain, staff-assessed pain, pain frequency, verbal descriptor of pain severity. These categorical variables were imputed relying on a discriminant function.

To select covariates to be included in the multiple imputation, preliminary analyses were conducted to evaluate variables that were predictive of pain with the expectation that identified predictors will provide the most amount of information in the imputation model and would capture indicators acting as a common cause between nursing home admission and pain and between cognitive impairment and pain. We relied on a-priori variable selection, manual review of cross tabulations, stepwise selection modeling. In addition to identifying predictive covariates, this step reduces the potential for inclusion of sets of covariates that are linear combinations of each other. Though linear combinations of covariates won’t impact the validity of pain imputations, their inclusion may adversely affect the run-time efficiency of the models. Candidate covariates were selected from MDS sections A (demographics and identifying information), D (mood assessments), G (functional status), I (active diagnoses), J (health conditions), L (oral and dental status), and M (skin conditions). Imputations were conducted separately for each state in order to (1) account for between state variability and (2) partition the data to run parallel imputations to improve run-time efficiency. The final set of covariates included in the imputation model is provided in table A1. SAS code for the imputation procedure is provided below.

| Supplemental Table 1: Listing of covariates included in the multiple imputation of pain metrics | | | | |
| --- | --- | --- | --- | --- |
| Covariates | |  | Covariates, continued | |
| Age | Continuous |  | Active Diagnoses | Cancer |
|  |  |  |  | Coronary artery disease |
| Gender | Men |  |  | Heart Failure |
|  | Women |  |  | Peripheral vascular disease |
|  |  |  |  | Cirrhosis |
| Race/Ethnicity | Asian |  |  | GERD |
|  | African American |  |  | Ulcerative Colitis |
|  | Hispanic |  |  | End Stage Renal Disease |
|  | White |  |  | Multidrug resistant organism |
|  |  |  |  | Pneumonia |
| Marital Status | Never Married |  |  | Tuberculosis |
|  | Married |  |  | Urinary Tract Infection |
|  | Widowed |  |  | Viral Hepatitis |
|  | Separated Divorced |  |  | Wound Infection |
|  |  |  |  | Diabetes |
| ADL Score | Continuous |  |  | Arthritis |
|  |  |  |  | Osteoporosis |
| Pain management | Scheduled pain medication |  |  | Hip Fracture |
|  | PRN pain medication |  |  | Other Fracture |
|  | Other pain intervention |  |  | Stroke |
|  |  |  |  | Hemiplegia or Hemiparesis |
| Dental | Cavity or broken teeth |  |  | Paraplegia |
|  | Mouth or facial pain |  |  | Quadriplegia |
|  |  |  |  | Multiple Sclerosis |
|  |  |  |  | Huntington's Disease |
| Skin conditions | Stage 1 or greater ulcer |  |  | Parkinson's Disease |
|  | Unhealed pressure ulcer |  |  | Seizure Disorder or Epilepsy |
|  |  |  |  | Traumatic Brain Injury |
| PHQ-9 | Lost Interest |  |  | Anxiety Disorder |
|  | Feeling down |  |  | Depression |
|  | Trouble sleeping |  |  | Manic Depression |
|  | Little energy |  |  | Psychotic Disorder |
|  | Poor apatite |  |  | Schizophrenia |
|  | Feels bad about self |  |  | PTSD |
|  | Trouble concentrating |  |  | Asthma, COPD, or CLD |
|  | Moving/Speaking Slowly |  |  | Respirator failure |
|  | Life not worth living |  |  | Cataracts, Glaucoma |
|  | Combined mood score |  |  |  |

SAS code for multiple imputation

**%macro** stateMI_Test (state, stateMI);

proc MI data = MI nimpute=5**0** out=tempMI;

where state_cd=&state;

class

/* For “Imp_comb_” variables, set to self-report if observed, otherwise set to staff assessed

for all “imp_” variables, set to missing of moderate or severe cognitive impairment*/

IMP_Comb_D0200A2_Intrst_Loss

IMP_Comb_D0200B2_Feel_Down

IMP_Comb_D0200C2_Trbl_Sleep

IMP_Comb_D0200D2_Ltl_Enrgy

IMP_Comb_D0200E2_Poor_Aptit

IMP_Comb_D0200F2_Self_Dprctn

IMP_Comb_D0200G2_Cncntrtn

IMP_Comb_D0200H2_Mvmt_Dfrnt

IMP_Comb_D0200I2_Ngtv_State

IMP_comb_anypain

IMP_J0400_Pain_Freq_Cd

IMP_J0600B_Vrbl_Dscrptr

IMP_pain_pre_stf

IMP_j0300_pain_cd

r_A1200_Mrtl_Stus_Cdn

;

| var  r_A1200_Mrtl_Stus_Cdn  ADLcompromise  C_RSDNT_AGE_NUM  r_A1000B_Asn_CdN  r_A1000C_Afrcn_Amrcn_CdN  r_A1000D_Hspnc_CdN  r_A1000F_Wht_CdN  r_A1200_Mrtl_Stus_CdN  r_I0100_Cncr_CdN  r_I0400_CAD_CdN  r_I0600_Hrt_Failr_CdN  r_I0900_PVD_CdN  r_I1100_Crrhs_CdN  r_I1200_GERD_CdN  r_I1300_Ulcrtv_Clts_CdN  r_I1500_ESRD_CdN  r_I1700_MDRO_CdN  r_I2000_Pneumo_CdN  r_I2200_TB_CdN  r_I2300_UTI_CdN  r_I2400_Vrl_Hpt_CdN  r_I2500_Wnd_Infctn_CdN  r_I2900_DM_CdN  r_I3700_Arthts_CdN  r_I3800_Ostprs_CdN  r_I3900_Hip_Frctr_CdN  r_I4000_Othr_Frctr_CdN  r_I4500_Strk_CdN  r_I4900_Hemiplg_CdN  r_I5000_Paraplg_CdN  r_I5100_Quadplg_CdN  r_I5200_MS_CdN  r_I5250_Hntgtn_CdN  r_I5300_Prknsn_CdN  r_I5400_Szre_CdN  r_I5500_Brn_Injury_CdN | r_I5700_Anxty_Dsordr_CdN  r_I5800_Dprsn_CdN  r_I5900_Mnc_Dprsn_CdN  r_I5950_Psychtc_CdN  r_I6000_Schzoprnia_CdN  r_I6100_PTSD_CdN  r_I6200_Asthma_CdN  r_I6300_Rsprtry_Failr_CdN  r_I6500_Ctrct_CdN  r_J0100A_Schld_Pain_Mdctn_CdN  r_J0100B_PRN_Pain_Mdctn_CdN  r_J0100C_Othr_Pain_Intrvtn_CdN  r_L0200D_Cvty_CdN  r_L0200F_Mouth_Pain_CdN  r_M0100A_Risk_Vsbl_CdN  r_M0210_Stg_1_Hghr_Ulcr_CdN  women  IMP_Comb_D0200A2_Intrst_Loss  IMP_Comb_D0200B2_Feel_Down  IMP_Comb_D0200C2_Trbl_Sleep  IMP_Comb_D0200D2_Ltl_Enrgy  IMP_Comb_D0200E2_Poor_Aptit  IMP_Comb_D0200F2_Self_Dprctn  IMP_Comb_D0200G2_Cncntrtn  IMP_Comb_D0200H2_Mvmt_Dfrnt  IMP_Comb_D0200I2_Ngtv_State  IMP_Comb_Mood_Scre  IMP_Comb_PainFreq  IMP_J0400_Pain_Freq_Cd  imp_j0300_pain_cd  IMP_pain_pre_stf  IMP_J0600A_Pain_Intnsty  IMP_J0600B_Vrbl_Dscrptr  IMP_Comb_AnyPain |
| --- | --- |

;

fcs discrim (

IMP_Comb_D0200A2_Intrst_Loss

IMP_Comb_D0200B2_Feel_Down

IMP_Comb_D0200C2_Trbl_Sleep

IMP_Comb_D0200D2_Ltl_Enrgy

IMP_Comb_D0200E2_Poor_Aptit

IMP_Comb_D0200F2_Self_Dprctn

IMP_Comb_D0200G2_Cncntrtn

IMP_Comb_D0200H2_Mvmt_Dfrnt

IMP_Comb_D0200I2_Ngtv_State

IMP_J0400_Pain_Freq_Cd

IMP_J0600B_Vrbl_Dscrptr

IMP_pain_pre_stf

imp_j0300_pain_cd

imp_comb_anypain

IMP_Comb_PainFreq

r_A1200_Mrtl_Stus_Cdn

/ classeffects=include) ;

run;

Data MIS.&stateMI; set tempMI;

run;

**%mend** stateMI;

%***stateMI*** (STATE='AK', stateMI=MI_AK)

%***stateMI*** (STATE='AL', stateMI=MI_AL)

%***stateMI*** (STATE='AR', stateMI=MI_AR)

%***stateMI*** (STATE='AZ', stateMI=MI_AZ)

%***stateMI*** (STATE='CA', stateMI=MI_CA)

%***stateMI*** (STATE='CO', stateMI=MI_CO)

%***stateMI*** (STATE='CT', stateMI=MI_CT)

%***stateMI*** (STATE='DC', stateMI=MI_DC)

%***stateMI*** (STATE='DE', stateMI=MI_DE)

%***stateMI*** (STATE='FL', stateMI=MI_FL)

%***stateMI*** (STATE='GA', stateMI=MI_GA)

%***stateMI*** (STATE='HI', stateMI=MI_HI)

%***stateMI*** (STATE='IA', stateMI=MI_IA)

%***stateMI*** (STATE='ID', stateMI=MI_ID)

%***stateMI*** (STATE='IL', stateMI=MI_IL)

%***stateMI*** (STATE='IN', stateMI=MI_IN)

%***stateMI*** (STATE='KS', stateMI=MI_KS)

%***stateMI*** (STATE='KY', stateMI=MI_KY)

%***stateMI*** (STATE='LA', stateMI=MI_LA)

%***stateMI*** (STATE='MA', stateMI=MI_MA)

%***stateMI*** (STATE='MD', stateMI=MI_MD)

%***stateMI*** (STATE='ME', stateMI=MI_ME)

%***stateMI*** (STATE='MI', stateMI=MI_MI)

%***stateMI*** (STATE='MN', stateMI=MI_MN)

%***stateMI*** (STATE='MO', stateMI=MI_MO)

%***stateMI*** (STATE='MS', stateMI=MI_MS)

%***stateMI*** (STATE='MT', stateMI=MI_MT)

%***stateMI*** (STATE='NC', stateMI=MI_NC)

%***stateMI*** (STATE='ND', stateMI=MI_ND)

%***stateMI*** (STATE='NE', stateMI=MI_NE)

%***stateMI*** (STATE='NH', stateMI=MI_NH)

%***stateMI*** (STATE='NJ', stateMI=MI_NJ)

%***stateMI*** (STATE='NM', stateMI=MI_NM)

%***stateMI*** (STATE='NV', stateMI=MI_NV)

%***stateMI*** (STATE='NY', stateMI=MI_NY)

%***stateMI*** (STATE='OH', stateMI=MI_OH)

%***stateMI*** (STATE='OK', stateMI=MI_OK)

%***stateMI*** (STATE='OR', stateMI=MI_OR)

%***stateMI*** (STATE='PA', stateMI=MI_PA)

%***stateMI*** (STATE='PR', stateMI=MI_PR)

%***stateMI*** (STATE='RI', stateMI=MI_RI)

%***stateMI*** (STATE='SC', stateMI=MI_SC)

%***stateMI*** (STATE='SD', stateMI=MI_SD)

%***stateMI*** (STATE='TN', stateMI=MI_TN)

%***stateMI*** (STATE='TX', stateMI=MI_TX)

%***stateMI*** (STATE='UT', stateMI=MI_UT)

%***stateMI*** (STATE='VA', stateMI=MI_VA)

%***stateMI*** (STATE='VT', stateMI=MI_VT)

%***stateMI*** (STATE='WA', stateMI=MI_WA)

%***stateMI*** (STATE='WI', stateMI=MI_WI)

%***stateMI*** (STATE='WV', stateMI=MI_WV)

%***stateMI*** (STATE='WY', stateMI=MI_WY)
